# The relationship between serum uric acid and colorectal cancer: a prospective cohort study

**DOI:** 10.1038/s41598-022-20357-7

**Published:** 2022-10-06

**Authors:** Wenqiang Li, Tong Liu, Sarah Tan Siyin, Qingsong Zhang, Yiming Wang, Liying Cao, Jun Qu

**Affiliations:** 1 Department of General Surgery, Aerospace Center Hospital, Yuquan Road 13, Haidian District, Beijing, 100038 China; 2 Department of Gastrointestinal Surgery/Clinical Nutrition, Capital Medical University Affiliated Beijing Shijitan Hospital, Beijing, 100038 China; 3 Department of General Surgery, Beijing Children’s Hospital, National Center for Children’s Health, Beijing, 100038 China; 4 Department of General Surgery, Kailuan General Hospital, Tangshan, 063000 China; 5 Department of Hepatobiliary Surgery, Kailuan General Hospital, Tangshan, 063000 China

**Keywords:** Gastrointestinal cancer, Tumour biomarkers

## Abstract

Serum uric acid (SUA) may play an important role in the occurrence of colorectal cancer (CRC). This study aims to explore the association of SUA with the risk of CRC incidence by drawing data from the Kailuan Study. We prospectively examined the association between SUA and risk of CRC incidence among 93,356 Chinese. Eligible participants were divided into three groups based on their tertiles of SUA. Cox proportional hazards regression was used to calculate the hazard ratios (HRs) and 95% confidence intervals (95% CIs) of CRC. During a median follow-up of 13.02 years, 583 new-onset CRC cases were identified. After adjustments were made for confounders, participants in the highest tertiles of SUA exhibited a 1.55-fold increased risk of CRC compared with patients with the lowest SUA levels (HR_T3 vs. T1_ = 1.55, 95% CI: 1.09–2.30). The associations of SUA with the risk of CRC were slightly reduced but remained substantial in the competing risk analyses when treating CRC unrelated death as the competing risk event. This study found a positive association of SUA with CRC incidence. Specific prevention efforts could be focused on the population with higher levels of SUA.

## Introduction

Colorectal cancer (CRC) rankse abdominal wall is a rare disease. We aim to elucidate the clinical and prognostic characteristics of this disease. Medical records of ten patients diagnosed with CCC of the abdominal wall at Fudan University Shanghai Cancer Center were reviewed. We illustrate the clinical characteristics, treatment modality, and development of local recurrence or distant metastasis, as well as the survival outcome. The median (range) age of patients was 47 (39–61) years old. All patients had a history of cesarean section and abdominal wall endometriosis. All patients had primary surgery before referred to our center. Seven patients had only tumor resection, while two patients had lymph node metastasis at primary diagnosis. Four patients underwent supplementary surgery, and all postoperative pathology were third in terms of global cancer incidence and represents 10% of all cancer cases^1,2^. It is the second leading cause of cancer-related deaths in the world. In 2020, there was an estimated 1.9 million new cases and 935,000 CRC-related death^3^. Despite CRC incidence and death rates stabilizing and even decreasing in developed countries in recent decades, low- and middle-income countries continue to struggle with the rapid growth of CRC^4–6^. China, the world’s fastest-growing developing country for the past four decades, has faced the impact of an aging population, urbanization, and a shift in lifestyle and diet changes which are both independently associated with CRC risk^4^. CRC incidence and its accompanying disease burden has rapidly increased in China and CRC is currently the country’s third most common cancer^7,8^.

Serum uric acid (SUA), a potent antioxidant, acts as a free radical scavenger^9^. The causative role of SUA in the development and progression of gout and metabolic syndrome is well recognized, and its associations with cardiovascular disease, acute ischemic stroke, and nonalcoholic fatty liver disease have been revealed recently^10,11^. However, evidence of a link between SUA and cancer risk has been heterogeneous, despite its antioxidant property^12^. Results from a Meta-analysis suggested that high SUA levels increased the risk of total cancer incidence and mortality^13^. Whereas, other studies failed to find such a relationship^14,15^. Metabolic factors such as SUA may play an important role in the occurrence of CRC. However, this association has not been explored in the prospective studies previously. This study aims to explore the association between SUA and the risk of CRC incidence by drawing data from the Kailuan Study (Trial identification: ChiCTR-TNRC-11001489; Registration number:11001489).

## Methods

### Study populations

The Kailuan Study, a prospective, ongoing cohort study in the Kaliuan community of Tangshan, China, was designed to explore the risk factors for chronic diseases, such as cancer. The study's details have been discussed previously^16^. In summary, 101,510 Kailuan Group employees (81,110 males and 20,400 women, ages 18 to 98) were invited to participate in the baseline health assessment between July 2006 and October 2007 and biannual follow-ups. At the baseline examination (2006–2007) and during each follow-up, each participant received standardized questionnaire evaluations, clinical assessments, and laboratory testing.

**Figure 1:**
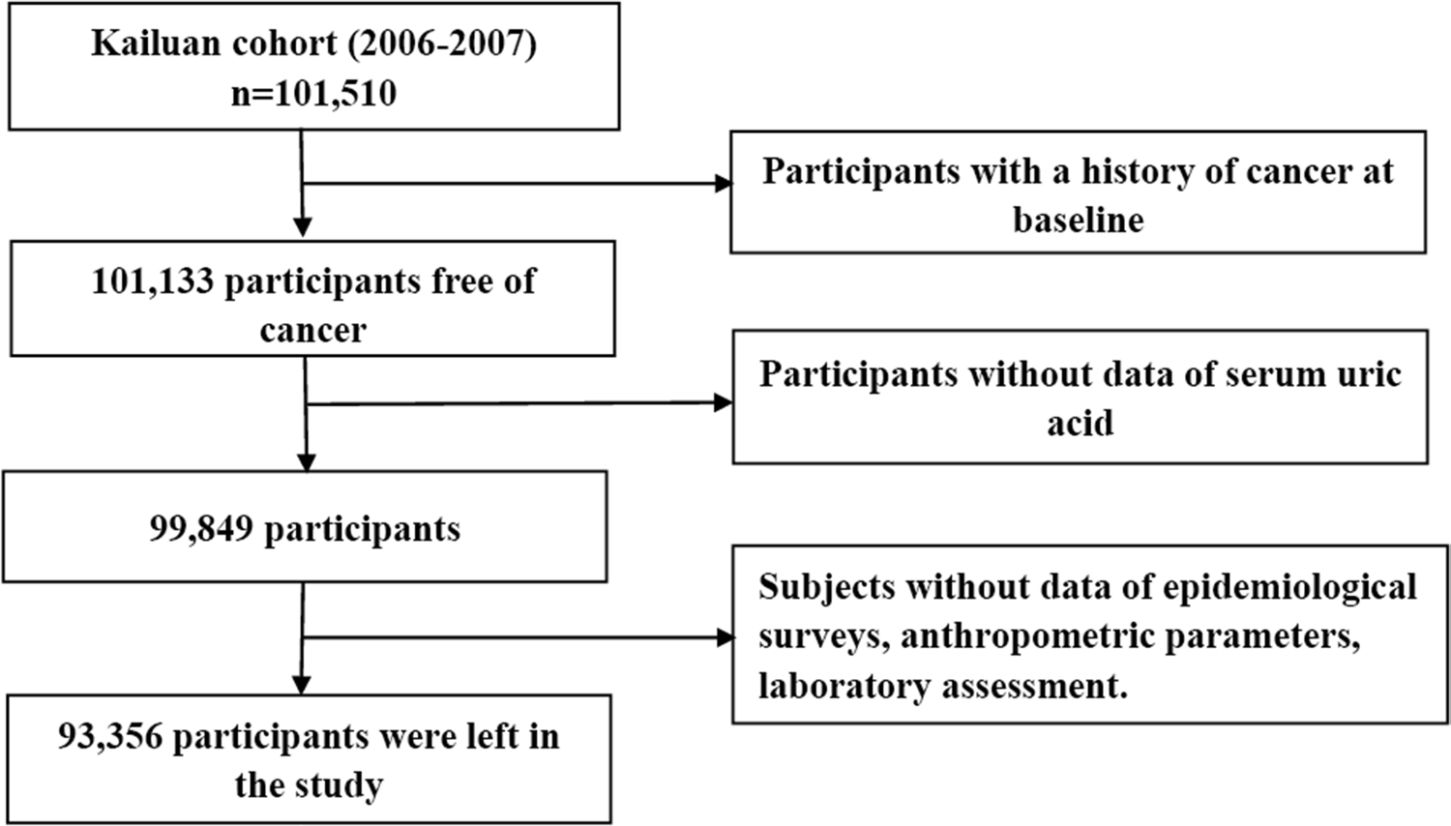
Flow chart of study participants.

We excluded 377 participants because they had a history of cancer at the time of the baseline assessment. We also excluded 1284 subjects who did not have SUA values. In addition, we excluded 6493 participants who did not have information on other potential confounders such as age, sex, body mass index (BMI, in kg/m^2^), systolic blood pressure (SBP, in mmHg), diastolic blood pressure (DBP, in mmHg), total cholesterol (TC, in mmol/L), triglyceride (TG, in mmol/L), high-sensitivity C-reactive protein (CRP, mg/L), socio-economic factors, and lifestyle behaviors. A total of 93,356 participants were finally enrolled in this study, including 18,826 women and 74,530 men (Fig. 1).

The protocol for this study was in accordance with the guidelines of the Helsinki Declaration and was approved by the Ethics Committee of Kailuan General Hospital and Aerospace Center Hospital. All the participants provided written informed consent.

### Assessment of serum uric acid

Blood samples were taken overnight (8–12 h) using vacuum tubes containing EDTA, then separated and kept at −80 °C for further analysis. The SUA concentrations were determined using an auto-analyzer (Hitachi 747; Hitachi, Tokyo, Japan) at Kailuan General Hospital's central laboratory using the oxidase technique. Males and females have different UA levels, and different cut-offs of tertiles were calculated for men (262.00, and 329.00 μmol/L) and women (211.00, and 267.00 μmol/L). Men and women were first separated into three groups based on sex-specific SUA tertiles and then pooled into: T1 group (n = 31,353), T2 group (n = 31,187), and T3 group (n = 30,816).

### Outcome ascertainment

The following methods were used to identify incident CRC cases: (1) reviewing clinical examinations participants underwent every two years until December 31, 2019, (2) checking medical records from the Tangshan medical insurance system and the Kailuan Social Security Information System yearly, and (3) reviewing death certificates from the Provincial Vital Statistics Offices (PVSO) once a year to obtain additional missing information. According to the International Classification of Diseases, Tenth Revision, clinical experts assessed the diagnosis and categorized CRC patients as C18-21 (ICD-10).

### Potential confounders

A standard questionnaire was used to collect information on each participant's age, sex, socioeconomic situation, educational background, living habits, and personal and family members' medical staff. The drinking and smoking status of participants was divided into four groups based on the information they provided in answer to the questions: never, past, moderate, and severe (having 100 ml/day of alcoholic beverages or smoked ≥ 1 cigarette/day)^17^. Physical activity was categorized as never, occasionally, or consistently (≥ 3 times/week, ≥ 30 min/time). The amount of time spent sedentary was divided into three categories: < 4 h/day, 4–8 h/day, and > 8 h/day. Tea consumption was divided into four categories: never, < 1 time/month, 1–3 times/month, 1–3 times/week, and > 4 times/week, as described previously^18^. The frequency of high-fat diets was divided into three categories based on the responses to the query. The estimated glomerular filtration rate (eGFR) was calculated by creatinine in line with the Chronic Kidney Disease Epidemiology Collaboration formula. Chronic Kidney disease was identified by either positive proteinuria or eGFR < 60 mL/ (min·1.73 m^2^)^19^. Family history of cancer was defined as at least one first-degree family member with cancer of any site.

Blood pressure (BP), height, and body weight were measured in standard methods by trained medical staff. BMI was calculated as the ratio of body weight (kg) divided by the square of height (m^2^) and classified into three groups: normal weight (< 24 kg/m^2^), overweight (24.00–27.99 kg/m^2^), or obese (≥ 28 kg/m^2^) based on the guideline of China Working Group on Obesity (WGOC)^20^. Hypertension was defined as having an SBP ≥ 140 mmHg or a DBP ≥ 90 mmHg, or a previous diagnosis.

The concentrations of TC, TG, and ALT were measured by the colorimetric enzymatic method (Mind Bioengineering Co. Ltd, Shanghai, China). Serum CRP was measured using a high-sensitivity nephelometry assay (Cias Latex CRP-H, Kanto Chemical Co. Inc, Tokyo, Japan). In accordance with the guidelines from the Centers for Disease Control and Prevention and the American Heart Association, CRP was divided into three groups: < 1 mg/L, 1–3 mg/L, and > 3 mg/L. The levels of serum TG, TC, ALT, and TBIL were divided into three groups by the tertiles of each variable. Diabetes mellitus was defined as follows: an FBG level ≥ 7.0 mmol/L, taking oral hypoglycemic agents or insulin, or a validated physician diagnosis^18^.

### Statistical analysis

Continuous variables were described as means ± standard deviations (SD) and comparisons were done with the one-way analysis of variance (ANOVA). The Chi-square test was used to compare categorical variables that were represented as absolute values with percentages. The person-year was determined from the date of the baseline examination through the date of the CRC diagnosis, death, or December 31, 2019, whichever came first. Restricted cubic spline regression (RCS) was used to calculate the dose–response relationship between SUA and CRC risks. The hazard ratios (HRs) and 95% confidence intervals (CIs) for the association of SUA with the risk of incident CRC were calculated using Cox proportional hazards models. Multivariate models were adjusted for the traditional risk factors of CRC including continuous variable (age) and categorical variables (sex, family income, educational background, marital status, BMI (< 24.00 kg/m^2^ vs. 24.00–27.99 kg/m^2^ and ≥ 28 kg/m^2^), tertiles of TG, TC, TBIL and ALT, CRP (< 1 mg/L vs. 1–3 mg/L, and > 3 mg/L), tobacco consumption, alcohol consumption, physical activity, sedentary lifestyle, tea consumption, high-fat diets, diabetes, hypertension, chronic kidney disease, and family history of cancer). The rationale for the selection of the confounders was based on the results from previous studies^21–23^.

During follow-up, cancer-unrelated death may occur before the occurrence of incident cancer, preventing us from identifying the presence of CRC. Because individuals with competitive events (and censored) are thought likely to develop cancer in the future, standard predictions such as Cox regression may overstate cancer risk in this competing risk setting. We further conducted the competing risk analyses including cause-specific hazards (CS) and sub-distribution hazard function (SD) models by fitting into the COX regression with a different definition of the event as follows: 0 = alive participants without CRC at the end of the study; 1 = participants diagnosed with CRC during follow-up; 2 = participants censored due to CRC unrelated death). Subgroup analyses were conducted by stratifying participants according to sex, age (≤ 45 years, 45–65 years, > 65 years), and BMI (< 24 kg/m^2^, 24–27.9 kg/m^2^, ≥ 28 kg/m^2^). Cancer patients were found to have higher SUA than healthy individuals^24^. Patients with occult malignancy or undiagnosed precancerous lesions at baseline sampling may cause reverse causation; we further excluded CRC cases that occurred within the first year or the first 5 years of follow-up in the sensitivity analysis.

A P-value (two-sided) < 0.05 was considered statistically significant. Statistical analyses were performed using a commercially available software program (SAS software, version 9.4).

### Ethics approval and consent to participate

This study was approved by the ethics committee of Kailuan General Hospital and Aerospace center hospital followed the Declaration of Helsinki. Informed consent forms were signed by the participants.

## Results

Table 1 summarizes the baseline characteristics of participants stratified by the tertiles of SUA. The mean age of the study population was 51.69 ± 12.48 years. Significant differences were found in age, levels of TC, TG, ALT, TBIL, hs-CRP and BMI, the percentages of educational background, marital status, reported income, physical exercise, sedentary lifestyle, tobacco consumption, alcohol consumption, tea consumption, high-fat diets, hypertension, diabetes mellitus, and family history of cancer among three prespecified SUA groups.

**Table 1:** Baseline characteristics of the participants stratified the tertiles of SUA.

Variables	T1 group	T2 group	T3 group	*P*-value
n (%)	31,353	31,187	30,816	
Age (year)	50.79 ± 10.17	51.51 ± 12.33	52.78 ± 13.13	< 0.001
Male (%)	25,083 (80.00)	24,830 (79.62)	24,617 (79.88)	0.327
**Reported income (¥)**				< 0.001
< 600	8633 (27.53)	9267 (29.71)	9034 (29.32)	
600–800	19,361 (61.75)	18,250 (58.52)	15,501 (50.30)	
800–1000	2143 (6.83)	2507 (8.03)	2487 (8.07)	
> 1000	1216 (3.87)	1163 (3.73)	3794 (12.31)	
**Marital status (%)**				< 0.001
Never	511 (1.62)	530 (1.70)	521 (1.69)	
Married	29,721 (94.79)	29,665 (95.12)	28,708 (93.16)	
Divorced	284 (0.91)	259 (0.83)	255 (0.83)	
Widowed	636 (2.03)	607 (1.95)	684 (2.22)	
Remarried	201 (0.88)	126 (1.03)	648 (2.10)	
**Educational background (%)**				< 0.001
Never	334 (1.07)	383 (1.23)	433 (1.41)	
Primary school	2963 (9.45)	2782 (8.92)	3237 (10.50)	
Middle school	22,478 (71.69)	22,463 (72.03)	19,811 (64.29)	
High school	3947 (12.59)	3782 (12.13)	4418 (14.34)	
College graduate or above	1631 (5.20)	1777 (5.70)	2917 (9.47)	
**TC (%)**				< 0.001
< 4.51 mmol/L	10,370 (33.07)	10,458 (33.53)	10,372 (33.66)	
4.51–5.34 mmol/L	10,694 (34.11)	10,622 (34.06)	9920 (32.19)	
> 5.34 mmol/L	10,289 (32.82)	10,107 (32.41)	10,524 (34.15)	
**TG (%)**				< 0.001
< 1.02 mmol/L	11,814 (37.68)	10,981 (35.21)	8574 (27.82)	
1.02–1.64 mmol/L	10,881 (34.70)	10,734 (34.42)	9322 (30.25)	
> 1.64 mmol/L	8658 (27.61)	9472 (30.37)	12,920 (41.93)	
**ALT (%)**				< 0.001
< 14.80 μ/L	10,570 (33.71)	10,366 (33.24)	10,188 (33.06)	
14.80–22.00 μ/L	11,581 (36.94)	9805 (31.44)	11,232 (36.45)	
> 22.00 μ/L	9202 (29.35)	11,016 (35.32)	9396 (30.49)	
**TBIL (%)**				< 0.001
< 10.70 μmol/L	11,795 (37.62)	9805 (31.44)	10,066 (32.66)	
10.70–13.90 μmol/L	10,695 (34.11)	11,182 (35.85)	8766 (28.45)	
> 13.90 μmol/L	8863 (28.27)	10,200 (32.71)	11,984 (38.89)	
**CRP (%)**				< 0.001
< 1 mg/L	18,993 (60.58)	17,921 (57.46)	15,505 (50.31)	
1–3 mg/L	6653 (21.22)	7690 (24.66)	9641 (31.29)	
> 3 mg/L	5707 (18.20)	5576 (17.88)	5670 (18.40)	
**BMI (%)**				< 0.001
< 24 kg/m^2^	17,224 (54.94)	14,655 (46.99)	10,717 (34.77)	
24–28 kg/m^2^	9993 (31.87)	11,016 (35.32)	12,270 (39.82)	
> 28 kg/m^2^	4136 (13.19)	5516 (17.69)	7829 (25.41)	
**Physical exercise (%)**				< 0.001
Never	2829 (9.02)	2745 (8.80)	2547 (8.27)	
Occasionally	24,916 (79.47)	24,129 (77.37)	21,557 (69.95)	
Regularly	3608 (11.51)	4313 (13.83)	6712 (21.78)	
**Smoking status (%)**				< 0.001
Never	20,311 (64.78)	19,668 (63.06)	15,913 (51.64)	
Past	1783 (5.69)	1902 (6.10)	1631 (5.29)	
Moderate	987 (3.15)	1478 (4.74)	827 (2.68)	
Severe	8272 (26.38)	8139 (26.10)	12,445 (40.38)	
**Drinking status (%)**				< 0.001
Never	20,477 (65.31)	19,473 (62.44)	15,205 (49.34)	
Past	1022 (3.26)	1150 (3.69)	1444 (4.69)	
Moderate	5388 (17.18)	5939 (19.04)	6551 (21.26)	
Severe	4466 (14.24)	4625 (14.83)	7616 (24.71)	
**Sedentary lifestyle (%)**				< 0.001
< 4 h/day	24,672 (78.69)	22,398 (71.82)	22,784 (73.94)	
4–8 h/day	5812 (18.54)	7299 (23.40)	7369 (23.91)	
> 8 h/day	869 (2.77)	1490 (4.78)	663 (2.15)	
**Tea consumption (%)**				< 0.001
Never	24,952 (79.58)	23,877 (76.56)	21,314 (69.17)	
< 1 time/month	1312 (4.18)	1367 (4.38)	1500 (4.87)	
1–3 times/month	1633 (5.20)	2003 (6.42)	2019 (6.55)	
1–3 times/week	1313 (4.19)	1597 (5.12)	1690 (5.48)	
> 4 times/week	2143 (6.84)	2343 (7.51)	4293 (13.93)	
**High-fat diets (%)**				< 0.001
Seldom	2511 (8.00)	2689 (8.62)	2681 (8.70)	
Occasionally	25,991 (82.90)	26,001 (83.37)	24,873 (80.71)	
Regularly	2851 (9.09)	2497 (8.01)	3262 (10.59)	
Chronic kidney disease (%)	1785 (5.69)	1692 (5.43)	1537 (4.99)	< 0.001
Family history of cancer (%)	1028 (3.28)	1133 (3.63)	1251 (4.06)	< 0.001
Diabetes mellitus (%)	2901 (9.25)	2711 (8.69)	2169 (7.04)	< 0.001
Hypertension (%)	12,341 (39.36)	12,999 (41.68)	13,090 (42.48)	< 0.001

*BMI* body mass index, *WC* waist circumference, *TC* total cholesterol, *CRP* C-reactive protein, *TG* triglyceride, *ALT* alanine aminotransferase, *TBIL* total bilirubin, *BMI* body mass index.

**Figure 2:**
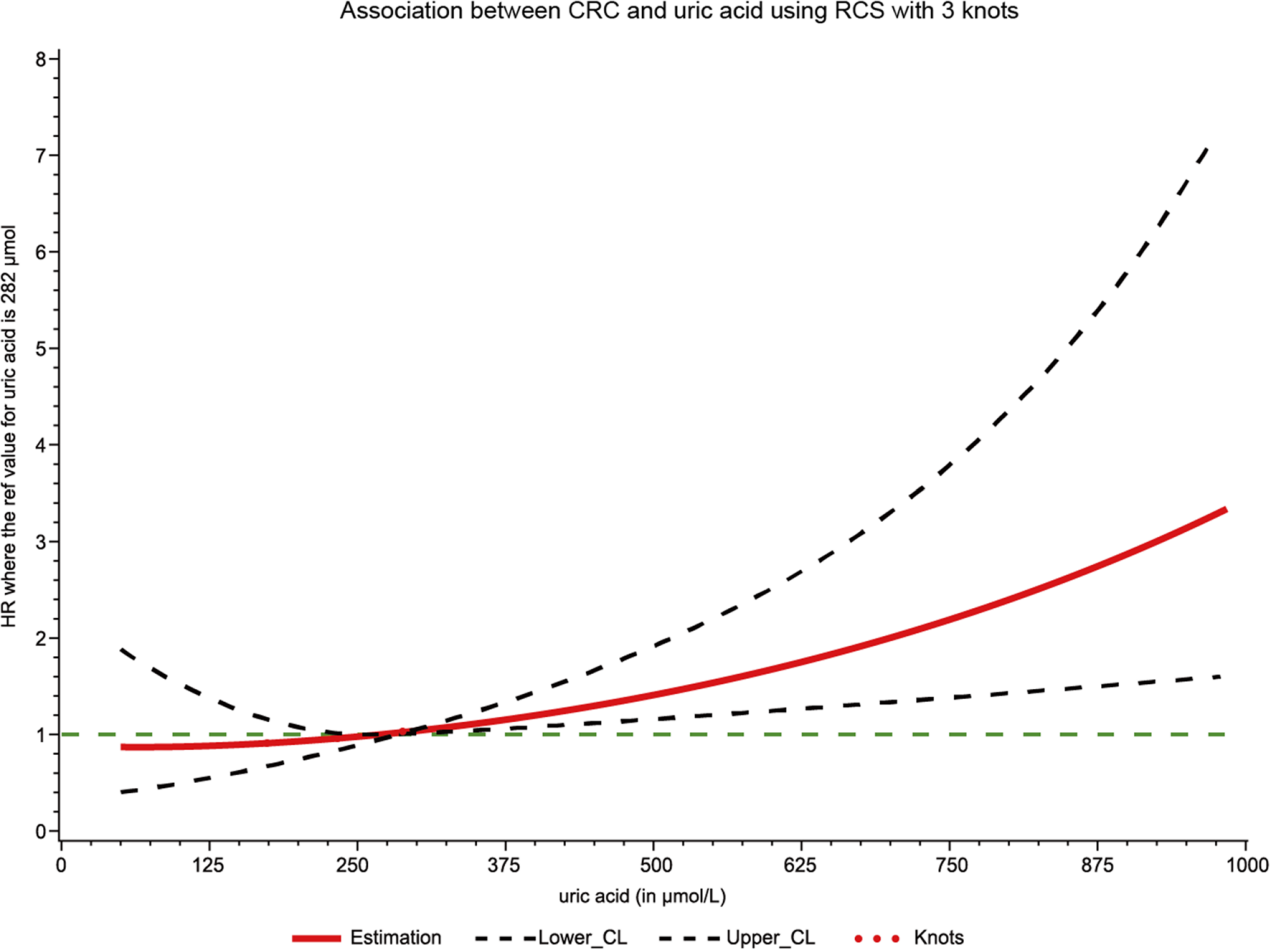
Association between SUA and CRC risk using RCS with 3 knots. Cubic spline graph of the adjusted HR (represented by solid line) and 95%CI (represented by the dotted lines).

The median (IQR) follow-up time was 13.02 (12.69, 13.20) years. A total of 583 new-onset CRC cases were diagnosed among 93,356 participants at the end of the study. The RCS model showed a positive dose–response and linear association between SUA levels and the risk of CRC (*p*-overall = 0.004, *p*-nonlinear = 0.055; Fig. 2). Table 2 presents the crude and adjusted HRs (95%CI) for the association between SUA and the risk of CRC incidence. Compared with patients with the lowest SUA levels (T1 group), participants in T2, and T3 groups had a 1.47-fold and 2.30-fold increased risk of CRC in the crude models (HR_T2 vs. T1_ = 1.47, 95% CI: 1.02–2.01; HR_T3 vs. T1_ = 2.30, 95% CI: 1.53–3.40). After adjusting for the confounders, participants with the highest levels of SUA exhibited an elevated risk of CRC incidence (HR_T3 vs. T1_ = 1.55, 95% CI: 1.09–2.30).

**Table 2:** Hazard ratios (HRs) for the association between SUA and the risk of CRC.

Group	Cases/person-years	Crude models		Adjusted models	
		HR (95%CI)	*p*-value	HR (95%CI)	*p*-value
**Traditional Cox models**					
T1 group	164/385,559	Ref		Ref	
T2 group	188/383,406	1.47 (1.02, 2.01)	0.044	1.13 (0.92, 1.67)	0.435
T3 group	231/373,106	2.30 (1.53, 3.40)	< 0.001	1.55 (1.09, 2.30)	0.019
*P* for trend			< 0.001		0.004
**SD models**					
T1 group	164/385,559	Ref		Ref	
T2 group	188/383,406	1.44 (0.99, 2.01)	0.057	1.07 (0.87, 1.56)	0.510
T3 group	231/373,106	2.29 (1.52, 3.40)	< 0.001	1.50 (1.05, 2.25)	0.029
*P* for trend			< 0.001		0.005
**CS models**					
T1 group	164/385,559	Ref		Ref	
T2 group	188/383,406	1.47 (1.02, 2.01)	0.045	1.13 (0.91, 1.67)	0.460
T3 group	231/373,106	2.29 (1.53, 3.39)	< 0.001	1.55 (1.09, 2.30)	0.020
*P* for trend			< 0.001		0.005

Models were adjusted for age (every 10 years), sex, family income, educational background, marital status, BMI, TG, TC, TBIL, ALT, CRP, smoking status, drinking status, physical activity, sedentary lifestyle, tea consumption, high-fat diet, diabetes, hypertension, chronic kidney disease, and family history of cancer.

SD models: the sub-distribution hazard function models.

CS models: the cause-specific hazards models.

During the follow-up period, a total of 8,182 individuals died before the occurrence of CRC. Compared with the lowest SUA level group (T1), the adjusted HRs (95%CI) for the association of high levels of SUA group (T3) with CRC risk was 1.50 (1.05, 2.25) in the SD models. Similar results were also observed in the CS models with minor differences. A positive trend was observed in all analyses (all p for trend < 0.05).

Table 3 displays the effect of serum SUA on the occurrence of CRC after stratifying the participants by sex, age, and BMI. Significant associations between SUA and CRC risk were observed among male, young, middle-aged, and normal-weight participants after adjusting for the aforementioned confounders. However, no significant association was found in women, the elderly, and participants who were overweight (including obese).

**Table 3:** Subgroup analysis of the association of SUA with the risk of CRC.

	T1 group		T2 group		T3 group	
	Case/person-years	Adjusted HRs (95% CI)	Case/person-years	Adjusted HRs (95% CI)	Case/person-years	Adjusted HRs (95% CI)
**Sex**						
Women^a^	22/78,877	Ref	30/79,804	1.07 (0.62, 1.87)	32/76,883	1.25 (0.72, 2.17)
Men^b^	142/306,682	Ref	158/303,602	1.11 (0.90, 1.38)	199/296,223	1.58 (1.10, 2.51)
*p* for interaction				0.962		
**Age (years)**						
Age ≤ 45	17/133,763	Ref	19/118,813	1.82 (0.96, 3.44)	21/108,964	2.00 (1.10, 3.64)
45 < Age ≤ 65	114/218,372	Ref	119/223,301	1.55 (0.87, 2.99)	147/201,233	1.76 (1.22, 2.14)
Age > 65	33/33,424	Ref	50/41,294	1.68 (0.64, 4.42)	63/62,909	1.70 (0.68, 4.26)
*p* for interaction				0.338		
**BMI (kg/m**^**2**^**)**						
BMI < 24	83/228,576	Ref	82/188,049	1.82 (0.96, 3.44)	83/132,987	1.98 (1.08, 3.60)
24 ≤ BMI < 28	63/125,979	Ref	70/137,704	1.20 (0.66, 2.20)	87/157,283	1.21 (0.69, 2.13)
BMI ≥ 28	18/31,004	Ref	36/57,653	1.68 (0.64, 4.42)	61/82,836	1.78 (0.73, 4.29)
*p* for interaction				0.951		

All models were adjusted for age, sex, BMI, TG, TC, CRP, TBIL, ALT, diabetes, hypertension, family income, educational background, marital status, salt consumption, current smoker, drinking status, physical activity, chronic kidney disease, and family history of cancer except the stratified factors.

^a^The cut-offs of the SUA among females were 211.00 and 267.00 μmol/L, respectively.

^b^The cut-offs of the SUA among males were 262.00 and 329.00 μmol/L, respectively.

In the sensitivity analysis, after excluding 28 and 285 CRC cases that had occurred within the first or the first five year of follow-up, the association remained significant after adjusting for potential confounders (Table 4).

**Table 4:** Sensitivity analysis after excluding participants who occurred cancer within the first year and the first five years of follow-up.

Groups	Cases/person-years	Adjusted models	
		HR (95%CI)	*p*-value
**Exclude CRC cases diagnosed within the first year of follow-up**			
T1 group	153/385,550	Ref	
T2 group	178/383,401	1.09 (0.88, 1.68)	0.655
T3 group	224/373,103	1.57 (1.11, 2.31)	0.014
P for trend			0.001
**Exclude CRC cases diagnosed within the first 5 year of follow-up**			
T1 group	68/385,252	Ref	
T2 group	90/383,063	1.22 (0.92, 2.39)	0.351
T3 group	140/372,806	2.01 (1.58, 2.89)	< 0.001
P for trend			< 0.001

Models were adjusted for age (every 10 years), sex, family income, educational background, marital status, BMI, TG, TC, TBIL, ALT, CRP, smoking status, drinking status, physical activity, sedentary lifestyle, tea consumption, high-fat diet, diabetes, hypertension, chronic kidney disease, and family history of cancer.

## Discussion

This is the first prospective cohort study that investigated the association of SUA levels with the risk of CRC incidence among 93,356 Chinese participants. Overall, participants with elevated levels of SUA exhibited a higher risk of CRC incidence. Significant associations were also observed among men, young (< 45 years), middle-aged (45 < age ≤ 65), and normal-weight (BMI < 24) participants after adjusting for confounders. Competing risk analysis and sensitivity analysis further validated the robustness of the main findings.

Results from previous studies partly supported the findings in the current study. Kim HJ et al. found that SUA was significantly associated with BMI, waist circumference, SBP, DBP, TC, TG, and HDL, all of which were CRC risk factors^25^. A large prospective cohort study found a positive association between SUA levels and the risk of overall and specific cancers^26^. Another study observed that high SUA levels increase the risk of both total cancer incidence and mortality^13^. In a study comparing different levels of SUA in CRC patients, Yan and Zhu found that CRC patients with hyperuricemia had poorer overall survival rates compared to those without high SUA^27^. Cetin et al. suggested that metastases developed in a shorter time in CRC patients with higher SUA^28^. When comparing metastatic and nonmetastatic CRC, Cheng Yuan et al. have observed that SUA might be a novel marker in assessing metastasis and have recommended its use to help determine prognosis^10^. Mao et al. measured SUA levels at the time of CRC diagnosis and concluded that it was particularly useful as a prognostic factor in stage II and III patients^29^.

SUA is recognized as both an antioxidant and a prooxidant, both of which may play a part in carcinogenesis^15,26^. In the normal range, SUA acts as a chelator of transitional metal ions and scavenges free radicals, which contributes to the total antioxidative aptitude of plasma^9^. It has been hypothesized that the fundamental scavenging action of circulating SUA limits neoplastic change and ultimately reduces the risk of cancer^30^. However, recent studies have found antioxidant properties of SUA inferior to that of hydrophilic vitamin C or hydrophobic vitamin E due to chemical structure, rendering it unlikely to play a crucial protective part in quenching oxygen radicals^30,31^. At higher levels, SUA is independently correlated with increased cancer mortality^27,32,33^. As a prooxidant, SUA assists tumorigenesis by entering normal cells and encouraging tumor cell proliferation, migration, and survival^26,29^. A prooxidant environment is advantageous for tumor cell growth, and oxidative stress is involved in the pathogenesis of CRC^29^.

There are ethnic differences in oxidative stress between the Chinese and Caucasian populations due to genotypic variations^34,35^. Using a high-fat meal, Chumjit et al. were able to show that Asians respond more sensitively, thus leading to greater levels of radical damage^34^. The genetic polymorphisms between DNA repair enzymes linked to the pathogenesis and development of CRC vary among ethnic populations^29,36^. Diet and genetics are crucial factors related to the development and prognosis of CRC, but there have been limited studies focused on the Chinese population in this regard. Our study offers a better understanding of how SUA acts as a risk factor for CRC among Chinese populations.

Similar to our study, Yan et al. found that high SUA levels elevated the risk of total cancer mortality for males but not females^13^. As it is known that males and older populations tend to have higher levels of SUA than their respective counterparts, they rationalized that the risk discrepancy might be due to varying saturation of SUA levels and metabolism of SUA^6,30,31,33,37^. However, this cannot be used to explain our findings, as younger and middle-aged participants also had a positive association. Further research on the metabolism of SUA and how it differs in different populations is needed to better understand its mechanism as a risk factor for CRC.

On the other hand, it has also been hypothesized that cancer itself could stimulate increased levels of SUA through cancer-related cell death rather than being an independent risk factor^33^. Cancer cachexia would lead to decreased fat stores and increased muscle wasting, which would in turn release glutamate and glutamine, both of which would increase SUA. A case-cohort sample including a random sub-cohort from Germany found no significant associations between uric acid and CRC^15^. Another study from Taiwan found that gout (a disease that is often linked with increased SUA) showed no significant association with an increased risk of CRC occurrence^38^. However, the former study used data from a Caucasian population and the latter did not adjust for other confounders.

Our study has several strengths. First, the prospective design of the Kailuan study and our large sample size (93,356 participants) add to the reliability of our findings. Second, the long-term follow-up allowed us to track the outcomes of participants for a longer period, thus limiting inaccuracy when estimating risk in the existence of competing events. Third, competing risks are frequent in epidemiologic research. The use of competing risk models (CS and SD models) allowed us to estimate individual risk as precisely as possible. This is especially evident in the presence of multiple competing events.

Limitations in the current study should also be noticed. First, SUA could, to a certain extent, vary during the follow-up period due to the single measurement, causing misclassification of participants. Second, our data was only collected from the Northern Chinese population, extrapolation to other populations would not be accurate. Third, participants who receive treatment for gout or hyperuricemia may have low SUA levels. The results may yield to the misclassification of the study population due to the limited data on the treatment.

## Conclusions

In this large-scale cohort, we found a positive association of SUA with the risk of incident CRC, especially among male, young- and middle-aged (≤ 65 years), and normal-weight (BMI < 24) participants. Future research should better elucidate the potential mechanisms of elevated levels of SUA for carcinogenesis. Importantly, specific prevention efforts could be focused on populations with higher levels of SUA.

## Data Availability

Data will be made available upon reasonable request (Jun Qu, E-mail: qujunchief@163.com).
